# Ghosts of weather past? Impact of past and present weather-related factors on the seasonal questing activity of *Ixodes ricinus* nymphs in southwestern Finland

**DOI:** 10.1186/s13071-025-06911-y

**Published:** 2025-07-14

**Authors:** Niko Tanski, Jari Hänninen, Risto Kalliola, Jani Jukka Sormunen

**Affiliations:** 1 Department of Geography and Geology, University of Turku, 20014 Turku, Finland; 2 Archipelago Research Institute, Biodiversity Unit, University of Turku, 20014 Turku, Finland; 3 Department of Biology, University of Turku, 20014 Turku, Finland; 4 Biodiversity Unit, University of Turku, 20014 Turku, Finland

**Keywords:** Ticks, Abiotic factors, Temporal tick dynamics, Longitudinal study, Time-lagged effects

## Abstract

**Background:**

Hard ticks are responsible for spreading several zoonotic infections globally. Of the main vector species in Europe, *Ixodes ricinus*, nymphal ticks cause the largest number of disease cases. Therefore, understanding the seasonal questing behaviour of this life stage is particularly crucial for public health. We assessed seasonal variation in questing abundance of *I. ricinus* nymphs on a tick-infested island in southwestern Finland. Our primary goal was to examine which abiotic factors, such as meteorological conditions from the recent past, influence the seasonal questing activity of *I. ricinus* nymphs, and whether these influences manifest similarly across different times and habitat types.

**Methods:**

Ticks were collected in 2012–2021 by cloth dragging from five different biotopes around the island. Three 50-m study transects were placed in each biotope, for a total of 15 transects. Air temperature and relative humidity were measured at the moment of sampling. Daily temperature and rainfall readings were obtained from weather stations.

**Results:**

Across all biotopes, the overall density of *I. ricinus* nymphs was 10.6 ticks/100 m^2^. In total, 7082 nymphs were collected from a total sampled area of 67,500 m^2^. Increasing nymph densities were observed during the 10-year study period, but the increase was not linear. Instead, an incremental jump in densities was observed in 2016. One weather-related explanatory factor remained in each of the statistical models for modelling the seasonal questing activity of ticks, when the progress of the season was already taken into account by week numbers.

**Conclusions:**

Increasing nymph densities were observed during a 10-year study period. While temperature measurements taken during the time of dragging did not appear to greatly influence the observed tick numbers, the recent past temperature variables were significant in all the natural biotopes. The results suggest that, in the clearly seasonal climate of southwestern Finland, the main factors shaping phenological patterns of *I. ricinus* nymphs during their main activity period are the progress of the season and a heat-related reduction in questing activity.

**Graphical Abstract:**

**Figure:**
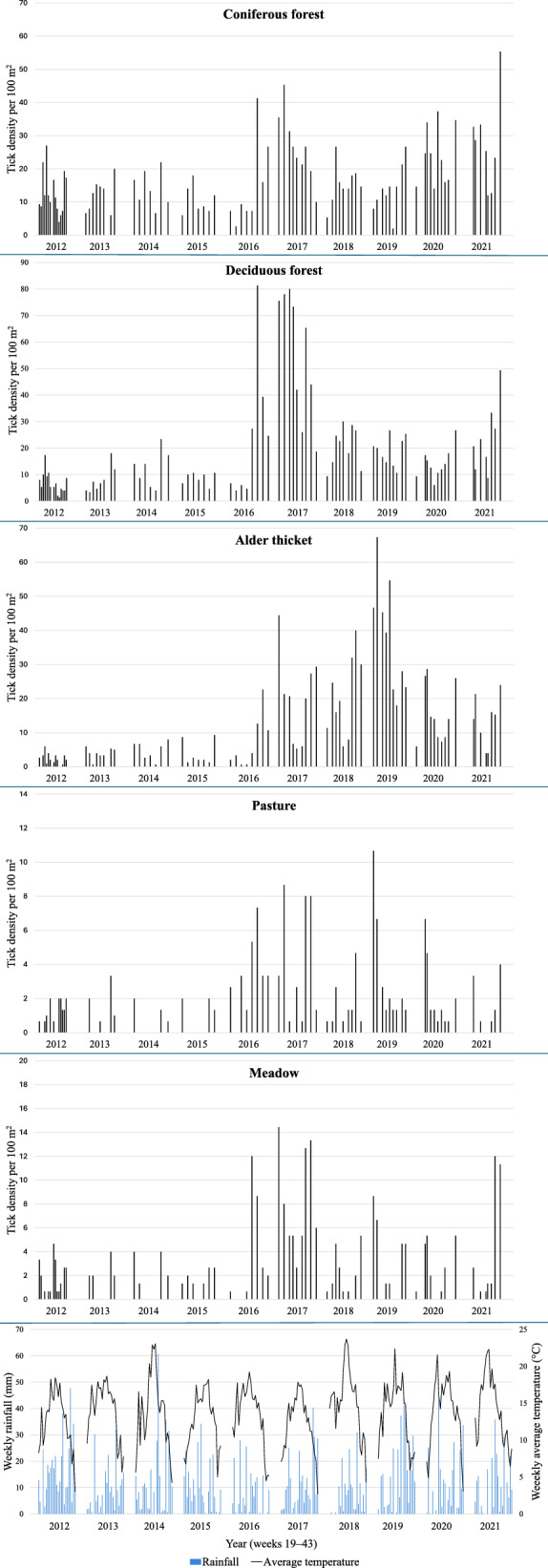

**Supplementary Information:**

The online version contains supplementary material available at 10.1186/s13071-025-06911-y.

## Background

Hard ticks are among the main vectors responsible for spreading several zoonotic infections globally [1, 2]. In the Northern Hemisphere, ixodid ticks are the primary vectors of several medically and veterinary relevant pathogens [3, 4]. Lyme borreliosis (LB) is the most frequently diagnosed tick-borne disease (TBD) in the Northern Hemisphere, with over 200,000 cases diagnosed in Europe each year [5, 6]. Humans acquire TBDs through the bites of infected ticks. Correspondingly, human risk is often measured with an entomologic risk index which incorporates tick abundance and prevalence rates of tick-borne pathogens in a single index [7]. For *Ixodes ricinus*, and correspondingly in the whole of Europe, nymphal ticks cause most disease cases. As such, risk is also measured using densities of *I. ricinus* nymphs [8]. Therefore, understanding the seasonal questing behaviour of this life stage in particular is crucial for public health.

Tick activity refers to the number of ticks that are actively seeking blood meals at a given time and place. Naturally, only active ticks attach to and bite humans. For *I. ricinus*, nymphs are the most dangerous tick life stage for humans, because they are small, hard to notice, and more numerous in nature than adult ticks, whilst already frequently carrying Lyme borreliosis spirochetes (*Borrelia burgdorferi *sensu lato) and tick-borne encephalitis virus (TBEV). In Finland, it is estimated that between 10% and 25% of questing nymphs carry *B. burgdorferi* (s.l.) [9]. Variation in tick activity is higher than that in *Borrelia* prevalence, and tick densities have correspondingly been observed to explain most of the variation in the entomologic risk index [10]. By understanding what causes variation in nymph activity, we can better assess disease risk and possibly target tick management efforts more precisely [11, 12]. The necessity for this approach has grown as observations made over the past few decades have revealed that the geographical distribution of *I. ricinus* has increased in Fennoscandia towards higher latitudes, accompanied by a substantial growth in abundance [13–18].

During the last three decades, the climate has become significantly warmer in Finland. For example, the average temperature for the entire year in Turku (Southwest Finland) was 6.2 °C between 1991 and 2020 [19]. In the years from 2010 to 2024, the annual average temperature was higher than 6.2 °C during all but four years [19]. Thermal growing seasons have become longer and warmer in Northern Europe [20]. According to a joint Nordic study, the reason for the lengthening of the growing season is particularly due to the earlier start of spring [20]. Warmer temperatures in spring and autumn lengthen the growing season for vegetation and extend the period during which *I. ricinus* as an ectothermic invertebrate can be active and develop.

In northern Eurasia, *Ixodes* ticks typically exhibit either unimodal or bimodal activity patterns during the warmer months of the year [21–24]. In Northern and Central Europe, tick activity usually follows a bimodal pattern, with one peak in the spring to early summer and another at the end of summer [25]. In Southern Europe, ticks have been observed to remain active and questing even during the winter months [26]. However, predicting ticks’ seasonal dynamics is challenging owing to the influence of various environmental factors on their abundance and questing behaviour, as well as their spatial variabilities across different geographical scales and habitat types. Ixodid ticks are impacted by weather conditions, especially temperature and relative humidity in their surroundings, both directly and indirectly [27–29]. Hot and dry conditions, in particular, raise tick death rates owing to increased climatic stress and prevent questing behaviour [30]. As a result, shifts in weather and climate will affect both the timing and length of the activity period of the ticks, as well as their geographical distribution, mortality, and developmental rates.

Studies have shown that factors such as temperature, rainfall and relative humidity can either increase or decrease the host-seeking activity of *Ixodes* ticks, depending on the habitat [31–35]. These factors are important mainly because they affect the tick’s water balance and energy use, which are critical for its survival [36]. Ticks may spend weeks or even months looking for a host, and when they start to lose too much water, they move to more humid spots to rehydrate. They will retreat to areas such as rotting plants in fields or damp leaves on a forest floor to stay hydrated. Earlier studies have found that measured temperature and humidity at the time of collection as well as a saturation deficit [37], an index combining temperature and humidity levels, can help explain some part of tick activity patterns throughout the year. However, these measurements only provide a momentary view of the situation. As a result, tick activity can also be observed in conditions clearly suboptimal for ticks, such as a relative humidity as low as 35% [38]. In reality, tick activity is also influenced by past weather conditions—for example, how much water has been lost in the previous days is likely to influence how long ticks can remain active in low-humidity conditions. The influence of past weather, “the ghost of weather past”, is more rarely in focus in studies of factors influencing tick activity. The incorporation of data on past weather is important for more accurate assessments that include the relevance of factors measured at the time of tick collection.

Recognizing both the significance and the poorly understood nature of weather-related factors on the phenology of *I. ricinus*, we undertook a 10-year study of tick questing activity on an island in southwestern Finland. The data on nymph questing activity are from regularly repeated linen cloth dragging conducted throughout the entire summer in five different habitats. The explanatory data used in this analysis are from daily measurements of precipitation and temperature, encompassing measurements both taken at the time of collection and calculated for different time lags. Our primary goal was to examine which abiotic factors, such as meteorological conditions from the recent past, influence the questing activity of *I. ricinus* nymphs, and whether these influences manifest similarly across different times and habitat types.

## Methods

### Field studies

**Fig. 1:**
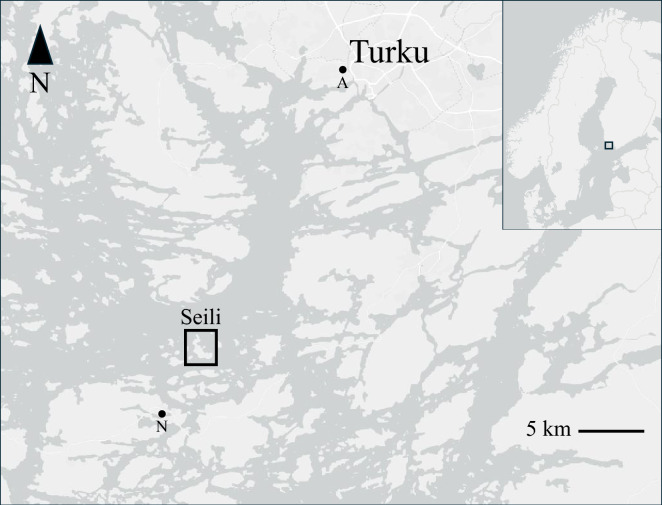
Study location. The location of the study area, Seili Island, in the Archipelago Sea, SW Finland. The black dots represent the locations of the weather stations outside the study area (A, Artukainen (Turku); N, Nagu). Map source: ESRI (available at: www.esri.com)

The Island of Seili (60°14′4ʺN, 21°57′7ʺE) is a rural island, covering an area of 1.6 km^2^, situated in the middle of the archipelago of southwestern Finland (Fig. 1). The island is located about 30 km southwest of the City of Turku. It belongs to the hemiboreal area of Finland. The island’s natural landscape consists of three main areas: a lush, herb-rich forest in the south, an open cultural landscape in the centre, and a rugged, conifer-dominated terrain in the north. The most unique habitats on Seili are the expansive hazelnut-rich forests in the south and the rocky grasslands in the central region. In Finland, many municipalities around the archipelago and along the coast are considered high-risk zones for TBEV. The national vaccination programme offers free TBE vaccination to those who have a municipality of residence in Finland and who have a permanent home or a holiday house on Seili [39].

This area of islands has a humid climate with a moderate annual precipitation of 491 mm and an average temperature of 7.1 °C (years 2007–2023, from the observation station of Nagu, 60° 11′ 23″ N, 21° 53′ 50″ E; [40]). Compared with the mainland, where precipitation reaches 693 mm and an average temperature is 6.8 °C (years 2007–2023, from the observation station of Turku Artukainen, 60° 27′ 15″ N, 22° 11′ 03″ E; [19]), the archipelago experiences less rainfall and slightly warmer conditions.

**Fig. 2:**
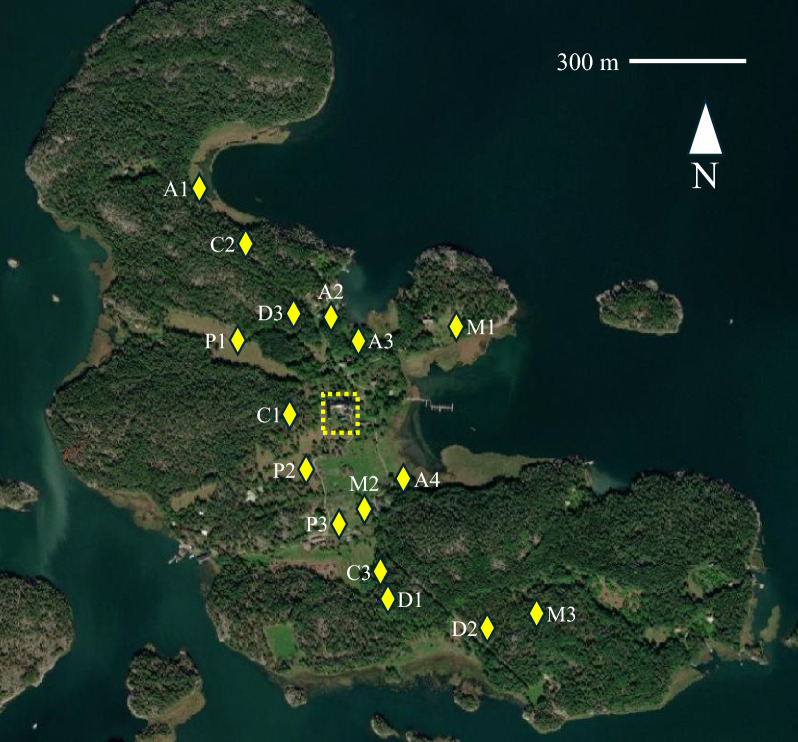
Study transects on the Island of Seili. Three transects were assigned to each biotope type, denoted with letter and number combinations. However, the transect A2 in an alder thicket needed to be moved permanently to another location (“A4”) in spring 2019. A, alder thicket; C, coniferous forest; D, deciduous forest; M, meadow; P, pasture. The dashed area indicates the location of the research institute, with the weather station on its roof. Map source: ESRI (available at: www.esri.com)

Between 2012 and 2021, field studies were carried out between one and three times a month during May–October. Ticks were sampled from five different habitats commonly found on the island: coniferous forest, deciduous forest, alder thickets, meadows and pastures. Ticks were gathered using cloth-dragging, which is the most widely used method for collecting and counting ticks. We dragged a white 1 × 1 m linen cloth across vegetation or a litter layer. Where possible, we avoided dragging across wet substrate and we did not sample in the rain, as this may lead to sampling bias [41]. Biotopes were classified as described in Ref. [16]. Three 50-m study transects were placed in each biotope, for a total of 15 transects. Descriptions of biotopes of these transects have been published in Ref. [42]. Generally speaking, “A” transects were found in areas dominated by common alders (*Alnus glutinosa*), “C” transects correspond to areas dominated by coniferous trees, “D” transects represent habitats dominated by deciduous trees or shrubs, “M” transects treeless habitats dominated by meadow foxtail (*Alopecurus pratensis*) and true grasses, and “P” transects treeless fenced grazing areas with flora similar to meadows. Cattle (since 2008) and sheep (since 2016) were brought to the island every spring to graze in rotation on various pastures across the island throughout the summer. One transect (“A2”) in an alder thicket needed to be moved permanently to another location (“A4”) in the spring of 2019 before the start of that year’s field studies, but otherwise the transects remained annually the same (Fig. 2).

Transects were divided into three roughly equal subsections, each measuring 16–17 m in length. These subsections were dragged individually to reduce the risk of ticks falling off during the process. After dragging each subsection, the cloth was thoroughly checked for ticks and the caught ticks were removed with tweezers. Each transect was dragged between one and three times a month. The total amount of dragging was 750–2250 m^2^ per month. Subsequently, each tick was counted by life stage, and a density of occurrence was determined, i.e., numbers per 100 m^2^.

Air temperature and relative humidity were measured at the moment of sampling. Measurements were taken about 10 cm above ground level using a handheld data logger (EL-USB-2-LCD RH/Temp data logger from DATAQ Instruments), since these variables are known to influence the questing behaviour of ticks [15, 43, 44].

Daily temperature and rainfall readings were obtained from a weather station (Davis Vantage Pro 2 Plus) situated in the middle of the island, on the roof of the Archipelago Research Institute (Fig. 2). The weather station has been operational since 2011. Using these measurements, both the average temperature and the amount of precipitation for the 1, 3, 5, 7, 9, 11, 14 and 28 days preceding the sampling were calculated. There was a fault in the weather station’s rain collector in 2017 and 2019–2021, and for these years the rainfall data were collected (by Davis Vantage Pro) from the Island of Storlandet (Nagu), 7 km away (Fig. 1) [45].

A significant amount of *Ixodes* ticks collected from Seili during this research period have been identified through either morphological or molecular methods, and *I. ricinus* is the only species that has been found [9, 16, 46].

### Statistical analyses

Original tick counts were used in statistical analyses. When measurements are taken repeatedly from the same individual, at the same place, or within the same time frame, they often show correlation [47]. This correlation can be managed by including random effects in generalised linear mixed models (GLMMs; [47]). We performed five separate GLMM data analyses for repeated measurements, as there were five different biotopes. This methodology is widely used especially in cases where a response variable, in general, is discrete or non-normally distributed. A response variable was the total number of nymphal ticks dragged from each transects. Since the tick data show great variability and are overdispersed, we used negative binomial models that are applied to model count data for which the variance is higher than the average. With these models, we also used log link functions, as we were using aggregated count data [48]. For descriptive purposes, we used a common density index (ticks/100 m^2^ of dragging).

As explanatory variables in our models, we used time trend (linear, quadratic, cubic or quartic polynomial for collection week to allow for unimodal or bimodal seasonal tick activity patterns), study transects and the interaction between these two, the collection year, the total amount of precipitation in 1, 3, 5, 7, 9, 11, 14 and 28 days and an average temperature of 1, 3, 5, 7, 9, 11, 14 and 28 days before the dragging day, a number of rainless days (precipitation < 0.2 mm/day) before the collection day, transect-specific temperatures and relative humidity measurements (at the time of dragging) and a saturation deficit index. Polynomials of a higher degree were chosen to be fitted into the model to reveal possible bimodality in the tick abundance from spring to autumn. Transects nested within the biotopes were the tested random variable for our models. To avoid problems of collinearity, the same types of weather-related variables were grouped into four distinct groups: (1) temperature, (2) rainfall, (3) relative humidity and (4) saturation deficit. From each group, only one variable at a time was included in the GLMM.

Analyses were performed using the residual pseudo-likelihood estimation technique of the GLIMMIX procedure of SAS 9.4. The higher degree of polynomials was tested as a single effect with multiple degrees of freedom using the NOSEPARATE option in the EFFECT statement. Denominator degrees of freedom were approximated by using the Kenward–Roger method [49].

## Results

**Fig. 3:**
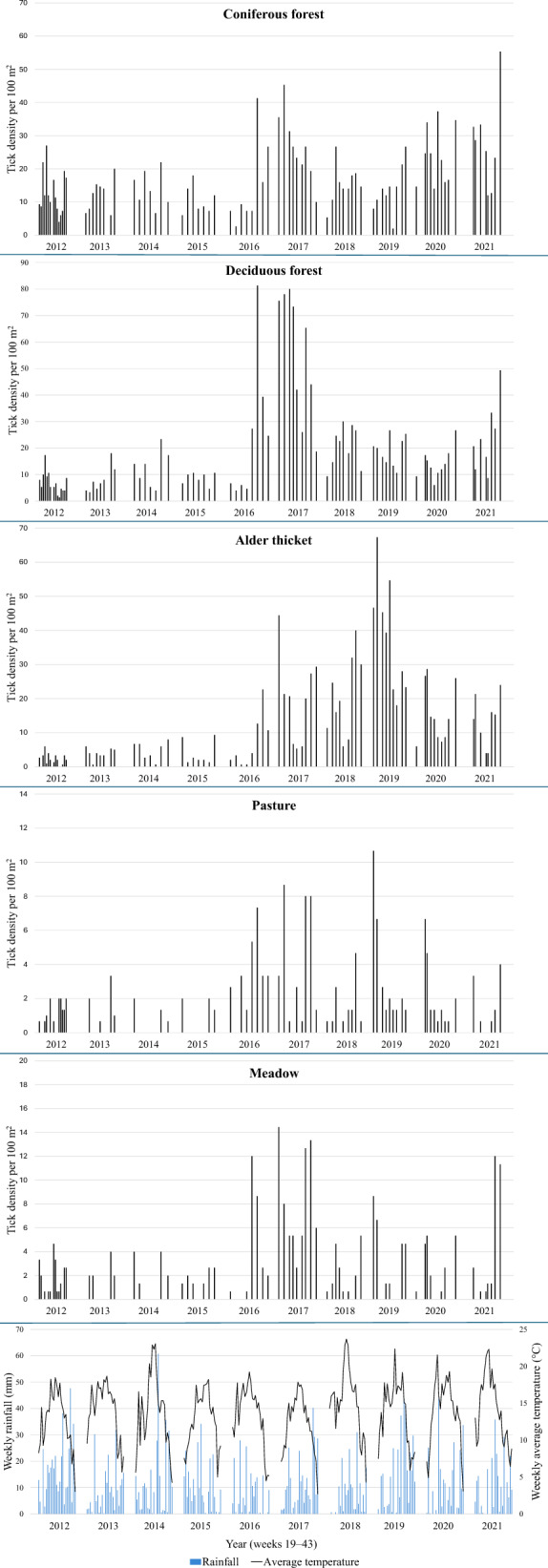
Weekly densities of *I. ricinus* nymphs (individuals per 100 m^2^) in all habitats on the Island of Seili, SW Finland. Data are shown by collection year (2012–2021), weekly rainfall and weekly average temperatures (weeks 19–43 each year). Note the different scales of *y*-axes, highlighting largely differing tick numbers among the biotopes

Weekly precipitation, average temperatures, and densities of nymphs in all habitats are shown in Fig. 3. Boxplots of temperatures and relative humidities at the time of dragging in all habitats are shown in Additional files 1: Fig. S1 and 2: Fig. S2. The highest amount of rain during weeks 19–43 was measured in 2012 (402.4 mm), while the lowest amount was in 2016 (177.4 mm). The highest average temperature during the same weeks of these years was in 2018 (15.4 °C), and the lowest was in 2017 (12.3 °C). In 2017, densities of nymphs in deciduous forests were also at their highest. Average temperatures and precipitation vary from year to year, but the changes do not fully seem to correspond with the variations in the density of nymphs (Fig. 3). In some years, a bimodal activity pattern is observed; hence we also applied polynomial time trends in our models. If the collections had been repeated every week in all years of study, this trend might have been observed even more clearly.

**Fig. 4:**
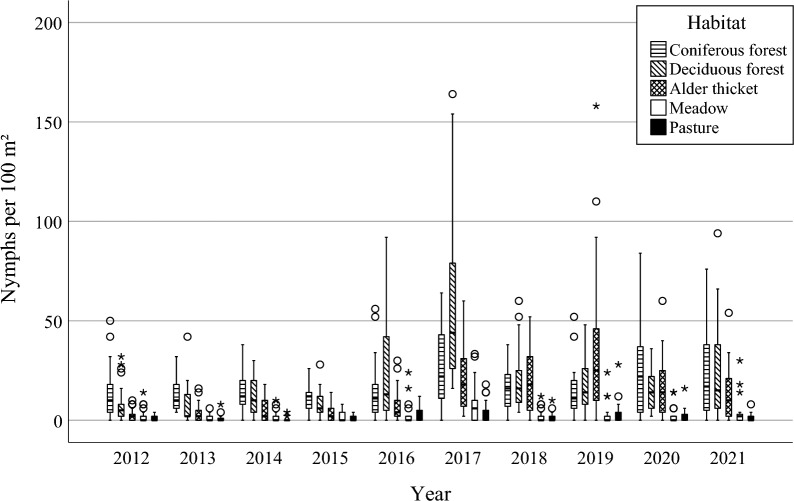
Boxplot of questing nymphal tick density per 100 m^2^ in all habitats. Shaded bars show the median and interquartile range of values for each index, while whiskers show minimum and maximum values; dots represent outliers and stars extreme outliers

Across all biotopes, the overall density of *I. ricinus* nymphs was 10.6 per 100 m^2^. In total, 7082 nymphs were collected from a total sampled area of 67,500 m^2^. However, tick densities varied significantly between biotopes. On average, transects in deciduous forests had the highest densities of host-seeking *I. ricinus* nymphs, but during the first years of research, the largest number of nymphs were found in coniferous forests (Table 1; Fig. 4). A considerable increase in tick density was also seen in alder thickets during the study period. As a general trend, yearly densities of nymphs rose during the study, though not in a linear manner.

**Table 1:** Densities of nymphs (per 100 m^2^) and averages of densities by year and by biotope

Biotope	2012	2013	2014	2015	2016	2017	2018	2019	2020	2021	Average per biotope
Coniferous forest	12.2	12.2	13.5	10.1	14.2	25.2	14.8	13.4	24.1	26.8	17.1
Deciduous forest	6.8	7.7	11.8	8.3	23.4	52.9	19.9	17.0	14.2	23.0	18.6
Alder thicket	2.1	3.8	4.6	3.8	7.1	18.3	20.1	34.0	15.9	13.0	12.8
Meadow	1.6	1.2	1.5	1.5	2.8	7.5	1.9	2.1	2.2	3.6	2.7
Pasture	0.9	0.8	0.5	0.7	3.2	3.6	1.4	2.8	2.1	1.2	1.8
Average per year	4.8	5.4	6.6	5.1	10.6	21.4	12.0	14.3	12.0	14.0	10.6

**Fig. 5:**
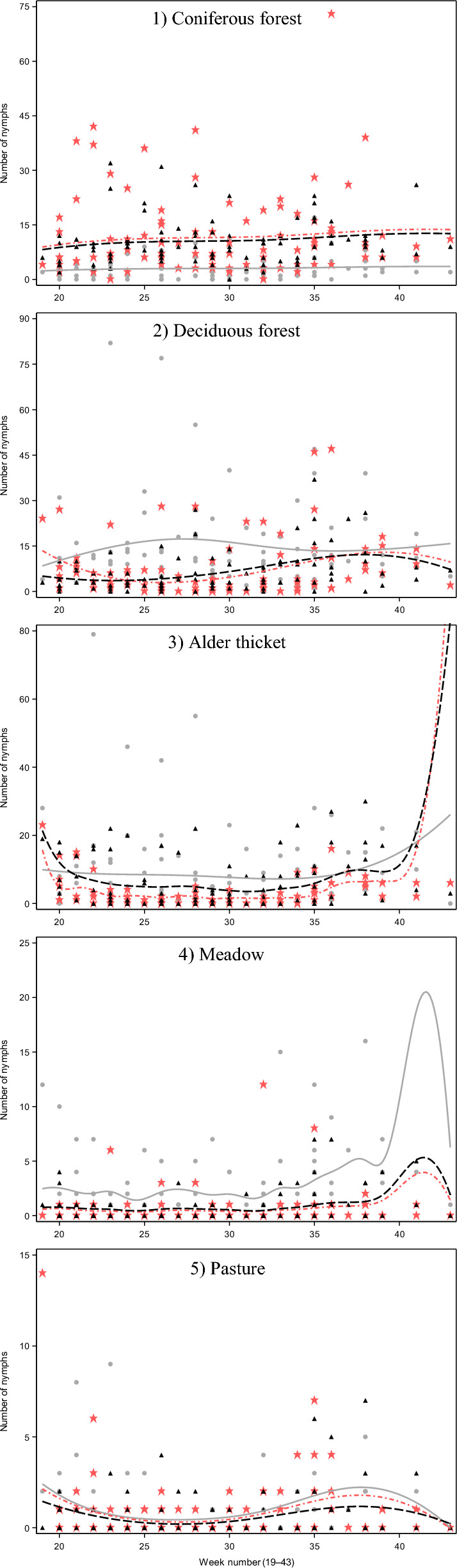
Sampling data (symbols) and polynomials predicting nymphal tick numbers (lines) in different biotopes (1–5). Owing to high variation even within biotopes, the prediction is made for each study transect separately (three transects per biotope), using the statistical models described in “Statistical analyses”. Note the different scales of *y*-axes, highlighting largely differing tick numbers among the biotopes

For the total number of nymphs in deciduous forests, the cubic time trend (week^3^) interacted significantly with the transect (Table 2). In alder thickets, there was an interaction between the transect and the quadratic time trend (week^2^). These indicated different questing activity patterns for nymphs in different transects of these biotopes (Fig. 5). According to values predicted by the GLMM, in one of the transects, a peak in tick questing activity was observed in early summer, while in the other transects, the peak was in late summer. The square time trend (week^2^) was strong in the statistical models for coniferous forests and meadows, and the quartic time trend (week^4^) in pastures.

**Table 2:** Results of generalised linear mixed models for the total number of nymphs for five different biotopes

Fixed effect – final model	Ndf	Ddf	*F*-value	*P*-value	Estimate	95% CI
(1) Coniferous forest						
Transect	2	253	12.93	< 0.0001		
Week^2^	2	253	74.92	< 0.0001		
Year	9	253	4.39	< 0.0001		
Temperature (°C), 14-day average	1	253	24.59	< 0.0001	–0.1314	[–0.184; –0.0791]
(2) Deciduous forest						
Transect	2	247	9.80	< 0.0001		
Week^3^	3	247	20.86	< 0.0001		
Transect × week^3^	6	247	6.59	< 0.0001		
Year	9	247	22.30	< 0.0001		
Temperature (°C), 14-day average	1	247	42.89	< 0.0001	–0.1729	[–0.225; –0.121]
(3) Alder thicket						
Transect	2	221.5	5.46	0.0048		
Week^2^	2	226.3	10.26	< 0.0001		
Transect × Week^2^	4	216	4.13	0.0030		
Year	9	247	24.65	< 0.0001		
Temperature (°C), 14-day average	1	247	21.28	< 0.0001	–0.1494	[–0.213; –0.0856]
(4) Meadow						
Transect	2	251	22.14	< 0.0001		
Week^2^	2	236.8	7.71	0.0006		
Year	9	251	2.87	0.0031		
Temperature (°C), 9-day average	1	251	12.31	0.0005	–0.1988	[–0.310; –0.0872]
(5) Pasture						
Transect	2	230.1	3.04	0.0496		
Week^4^	4	212.9	7.72	< 0.0001		
Year	9	240	4.66	< 0.0001		
Relative humidity (%), during dragging	1	216.3	4.04	0.0458	0.01790	[0.000341; 0.0355]

Parameter estimates (in log scale) of linear continuous factors are given in columns “Estimate” and “95% CI”

*Ndf* numerator degrees of freedom, *Ddf* denominator degrees of freedom

**Fig. 6:**
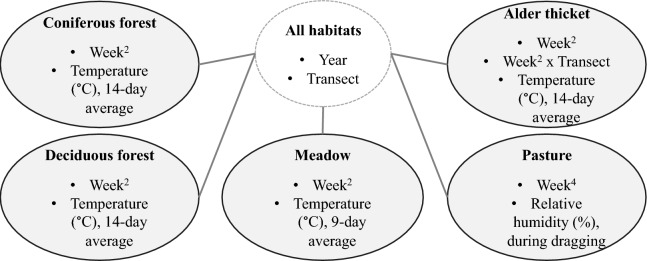
Visualised results of generalised linear mixed models for the total number of nymphs for five different biotopes. The year and transect were common explanatory factors in all models. Other explanatory variables for each model are shown inside the grey ovals

One weather-related explanatory factor remained in each of the final models when the progress of each season, i.e., increasing average temperatures from May towards July (spring to summer) and declining temperatures towards October thereafter (summer to autumn), was taken into account by week numbers (Table 2). The study year was included and significant in all models. In forested biotopes (coniferous and deciduous forests and alder thickets), higher average temperatures for the previous 14 days predicted lower numbers of nymphs, whereas in meadows higher average temperatures for the past 9 days had a similar negative effect. Higher relative humidity predicted higher numbers of nymphs in pastures. The results of five models are visualised in Fig. 6.

## Discussion

Increasing nymph densities were observed during the 10-year study period, but the increase was not linear. Instead, an incremental jump in densities was observed in 2016, followed by relatively stable annual densities at this new higher level (with 2017 a clear outlier). Similar jumps in densities were also observed between 2015 and 2017 on two islands less than 30 km from Seili, viz. Hirvensalo and Ruissalo (unpublished own data). Incremental rather than equal changes could signify that the cause is some unidentified threshold having been crossed, since densities were relatively stable before and after the jump. One possible explanation could be that the earlier springs and warmer early summers allow ticks finding an early blood meal to develop into the next life stage and obtain a second blood meal during the same summer, which would drastically reduce the life cycle duration and increase the reproductive potential of ticks [50]. While this subject has not been studied in natural conditions, there have been recent reports of adult *Hyalomma marginatum* being detected in northern parts of Europe (for example, in 2018 in Sweden and Great Britain [51, 52]; an adult was also detected in Helsinki, Finland, in 2024 (unpublished own data)). In the past, temperatures in these areas have not allowed *H. marginatum* nymphs arriving on migrating birds to moult into adults before the onset of colder weathers in autumn/winter; this suggests that changes in temperatures at ranges and scales relevant for tick biology are occurring [51, 52].

In addition to changes in abiotic conditions, also changes in host animal occurrence and abundance are likely to influence local tick densities. Several suitable host animals of different sizes inhabit Seili Island (for a more detailed description of local fauna, see Ref. [9]), but precise data on their occurrence are not available. Local hunters have anecdotally reported an increase in roe deer (*Capreolus capreolus*), white-tailed deer (*Odocoileus virginianus*) and raccoon dog (*Nyctereutes procyonoides*) populations on Seili during the past decade. All these species are important hosts for adult ticks, so increases in their availability may likewise be driving increasing tick populations. Indeed, both biotic and abiotic factors are likely behind the increased tick populations on the island.

Apart from annual changes in tick densities being apparent, the collection week also remained a significant variable in all the models, indicating differences in tick questing activity throughout the collection period. In this context, it is worth noticing that, in Northern Europe, the progress of the season also has a high correlation with the measured temperatures, with temperatures roughly forming a down-facing parabola from May to October [53]. This may explain why temperature measurements taken at the time of dragging did not endure in the models—the variation in current temperatures was already explained by the weekly temporal variable. Likewise, it is important to note that our collection period encompassed only the main activity period of *I. ricinus* in Finland from May to October—as such, no temperatures below the 5 ℃ tick activity threshold were observed during spring collections and only occasionally in late autumn. Consequently, questing activity was not restricted by cold temperatures, limiting the impact of current temperatures on activity [38, 53, 54]. Nonetheless, tick activity may also be influenced by temporal factors other than temperatures—for example, the activity of *I. persulcatus* by photoperiod [55]—so both types of variables should be included in models seeking to explain variation in observed tick activity.

While temperature measurements taken during the time of dragging did not appear to greatly influence observed tick numbers, past temperature variables were significant in all the natural biotopes. These associations were observed to be negative in all cases; i.e., the higher the temperatures during the past 9 or 14 days, the fewer ticks observed to be active. These negative associations are likely a reflection of periods of high temperatures and drought observed during most summers, typically in June or July in Finland. High temperatures and low relative humidity cause questing ticks to lose water at a rapid rate, eventually forcing them to retreat to more humid microhabitats to rehydrate and enter quiescence to await more facilitating questing conditions [37]. The need to rehydrate may mean that ticks are not immediately reactivated after individual rains or days of lower temperatures, potentially causing a lingering effect. For forested biotopes, temperature averages from the past 14 days persisted in the models, whereas for open/only partially shaded meadows the averages from the past 9 days remained. This indicates that ticks in shaded biotopes are somewhat more sheltered from the negative impacts of high temperatures and drought than those in open habitats. Trees provide shade through canopy cover, litter to form humid microhabitats at the ground level and cover from wind, all of which may lead to these habitats being able to buffer against desiccating conditions for longer periods than open habitats.

No temperature variables remained in the model for pastures. However, in pastures, the grazing of cattle dramatically alters the vegetation and microhabitats at random points during the summer (rotational grazing), which may be expected to also alter the natural responses of ticks to environmental conditions. Pasture transects had the lowest nymph densities observed, and it is indeed unclear whether the habitats covered by our pasture transects can support tick populations, or whether the ticks detected are occasional stragglers transported as feeding ticks from more forested parts of the pasturage to these open areas, where only some engorged individuals survive to the next life stage. While an overabundance of host animals in a given area (such as a herd of cattle) may reduce the numbers of ticks caught by cloth dragging owing to ticks readily finding hosts, in this case the cattle are only present at each pasture for roughly a month during the summer, leaving the areas with scarce medium/large hosts for most of the activity season. As such, if ticks that have successfully fed on the cattle were surviving transstadial development in the area, one would expect to find an abundance of larvae, nymphs and/or adults seeking blood meals during the other months. Nevertheless, the differing results from pastures compared with the natural biotopes may likely have no more significant meaning than that they are an indication of disturbed habitats.

Past rainfall and the number of rainless days before the collection day were observed to have no measurable impact on the numbers of questing nymphs. While rainfall patterns at the annual and seasonal scale influence tick survival and abundance [56], it is not clear how rain affects tick questing activity, as the standard collection methods of cloth dragging and flagging cannot be utilised in rain. However, it has been reported on the basis of experimental studies that ticks avoid liquid water [57]. It seems likely that a light drizzle will not immediately cause ticks to stop questing, whereas heavy rain might lead to a rapid retreat to cover. The impact of rain is certainly also influenced by the presence of canopy cover, with light rain possibly not affecting tick questing at all in areas with extensive cover—or even positively owing to increased relative humidity. Furthermore, a major difference between past temperatures and rainfall is that, unlike periods of extreme temperatures, even longer periods of rainy weather are likely to have no long-term or lingering impact on activity. Rain or wet ground has not been observed to cause quiescence, and ticks might in fact even be able to rehydrate while sheltering. As such, they may be ready to resume questing as soon as the ground/vegetation is sufficiently dry. Relative humidity remains high on the ground floor after rains as well, so questing conditions are possibly improved afterwards, also suggesting that any potential activity reducing effects are probably not persistent.

## Conclusions

We examined *I. ricinus* nymph seasonal questing activity over a 10-year study period on an island in southwestern Finland, and how this activity is influenced by past (here up to 28 days prior to collection) and present (at the time of collections) abiotic conditions. We showed increasing nymph densities in all habitats on the island, but the increase was not linear. Instead, an incremental jump in densities was observed in 2016, followed by relatively stable annual densities at this new higher level. Explanatory variables related to past temperatures were significant in all the natural biotopes, indicating that, the higher the temperatures of the previous 9–14 days, the lower the nymph questing activity. Past rainfall or the number of rainless days prior to collections was observed to have no measurable impact on the numbers of questing nymphs. The results of the present study suggest that, in the clearly seasonal climate of southwestern Finland, the main factors shaping phenological patterns of *I. ricinus* nymphs during their main activity period are the progress of the season, i.e., increasing average temperatures from May towards July (spring to summer) and declining temperatures towards October thereafter (summer to autumn), and a heat-related reduction in questing activity.

## Supplementary Information

Supplementary material 1.

Supplementary material 2.

## Data Availability

Data supporting the main conclusions of this study are included in the manuscript.
