# Supplementary material for: Ghosts of weather past? Impact of past and present weather-related factors on the seasonal questing activity of Ixodes ricinus nymphs in southwestern Finland
Source: Parasit Vectors. 2025 Jul 14;18:283. doi: 10.1186/s13071-025-06911-y (PMC12261623; doi:10.1186/s13071-025-06911-y)
Supplement: Supplementary file 2 — Supplementary material 2. [file 13071_2025_6911_MOESM2_ESM.pdf]

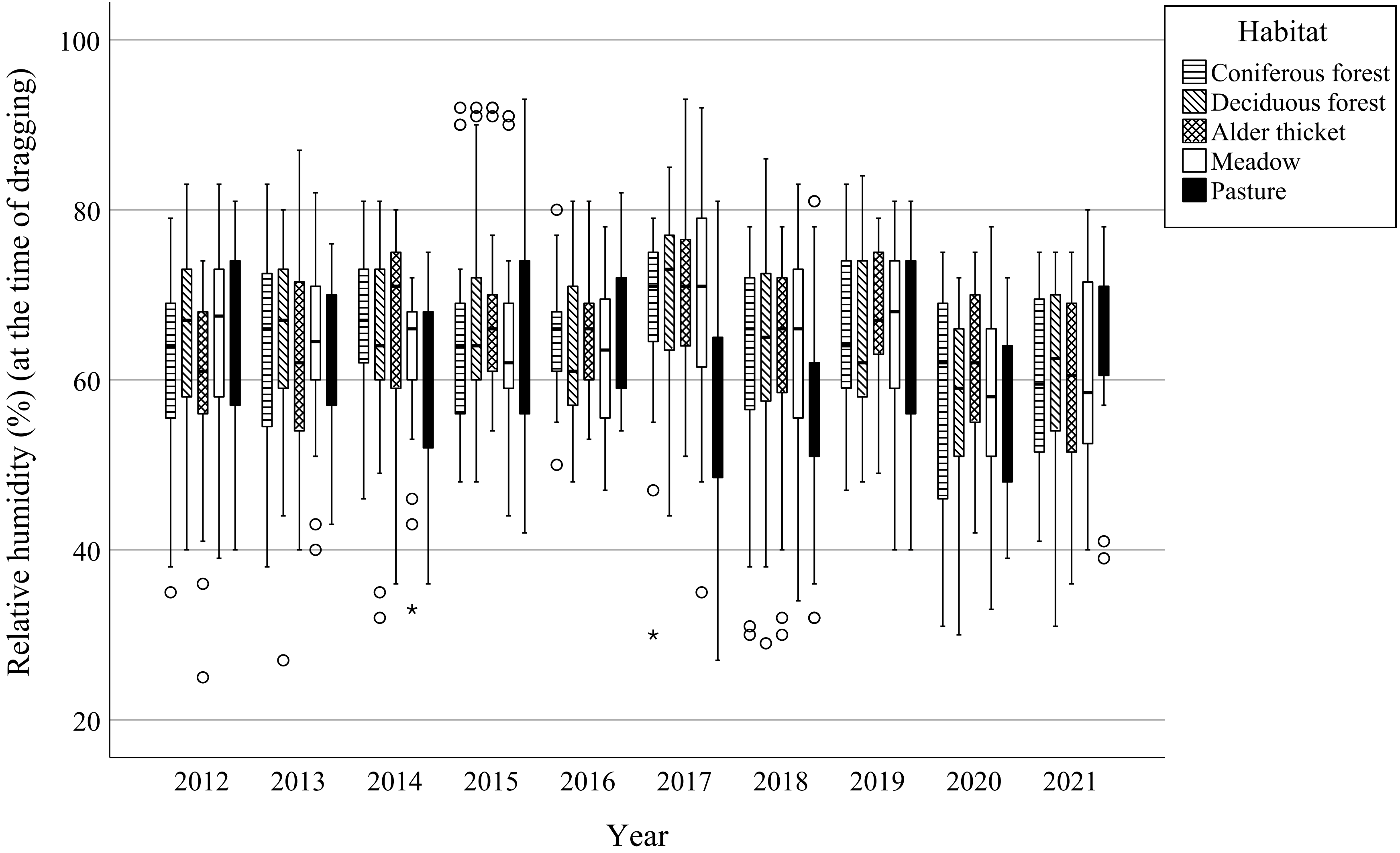

Habitat
Coniferous forest
Deciduous forest
Alder thicket
Meadow
Pasture
Relative humidity (%) (at the time of dragging)
100
80
60
40
20
2012
2013
2014
2015
2016
2017
2018
2019
2020
2021
Year
